# Home-based transcranial direct current stimulation plus tracking training therapy in people with stroke: an open-label feasibility study

**DOI:** 10.1186/s12984-018-0427-2

**Published:** 2018-09-18

**Authors:** Ann Van de Winckel, James R. Carey, Teresa A. Bisson, Elsa C. Hauschildt, Christopher D. Streib, William K. Durfee

**Affiliations:** 1 Division of Physical Therapy; Division of Rehabilitation Science, University of Minnesota, 420 Delaware Street SE (MMC388), Minneapolis, MN 55455 USA; 2 Division of Physical Therapy, University of Minnesota, Minneapolis, MN 55455 USA; 3 Department of Mechanical Engineering, University of Minnesota, Minneapolis, MN 55455 USA; 4 Department of Neurology, University of Minnesota, Minneapolis, MN 55455 USA

**Keywords:** Stroke, Neurological rehabilitation, Telerehabilitation, Transcranial direct current stimulation, Physical therapy

## Abstract

**Background:**

Transcranial direct current stimulation (tDCS) is an effective neuromodulation adjunct to repetitive motor training in promoting motor recovery post-stroke. Finger tracking training is motor training whereby people with stroke use the impaired index finger to trace waveform-shaped lines on a monitor. Our aims were to assess the feasibility and safety of a telerehabilitation program consisting of tDCS and finger tracking training through questionnaires on ease of use, adverse symptoms, and quantitative assessments of motor function and cognition. We believe this telerehabilitation program will be safe and feasible, and may reduce patient and clinic costs.

**Methods:**

Six participants with hemiplegia post-stroke [mean (SD) age was 61 (10) years; 3 women; mean (SD) time post-stroke was 5.5 (6.5) years] received five 20-min tDCS sessions and finger tracking training provided through telecommunication. Safety measurements included the Digit Span Forward Test for memory, a survey of symptoms, and the Box and Block test for motor function. We assessed feasibility by adherence to treatment and by a questionnaire on ease of equipment use. We reported descriptive statistics on all outcome measures.

**Results:**

Participants completed all treatment sessions with no adverse events. Also, 83.33% of participants found the set-up easy, and all were comfortable with the devices. There was 100% adherence to the sessions and all recommended telerehabilitation.

**Conclusions:**

tDCS with finger tracking training delivered through telerehabilitation was safe, feasible, and has the potential to be a cost-effective home-based therapy for post-stroke motor rehabilitation.

**Trial registration:**

NCT02460809 (ClinicalTrials.gov).

## Background

Post-stroke motor function deficits stem not only from neurons killed by the stroke, but also from down-regulated excitability in surviving neurons remote from the infarct [1]. This down-regulation results from deafferentation [2], exaggerated interhemispheric inhibition [3], and learned non-use [4]. Current evidence suggests that post-stroke motor rehabilitation therapies should encourage upregulating neurons and should target neuroplasticity through intensive repetitive motor practice [5, 6]. Previously, our group has examined the feasibility and efficacy of a custom finger tracking training program as a way of providing people with stroke with an engaging repetitive motor practice [7–9]. In this program, the impaired index finger is attached to an electro-goniometer, and participants repeatedly move the finger up and down to follow a target line that is drawn on the display screen. In successive runs, the shape, frequency and amplitude of target line is varied, which forces the participant to focus on the tracking task. In one study, we demonstrated a 23% improvement in hand function (as measured by the Box and Block test; minimal detectable change is 18% [10]) after participants with stroke completed the tracking training program [9]. While our study did not evaluate changes in activity in daily life (ADL) or quality of life (because efficacy of the treatment was not the study objective), the Box and Block test is moderately correlated (*r* = 0.52) to activities in daily life and quality of life (*r* = 0.59) [11]. In addition, using fMRI, we showed that training resulted in an activation transition from ipsilateral to contralateral cortical activation in the supplementary motor area, primary motor and sensory areas, and the premotor cortex [9].

Recently, others have shown that anodal transcranial direct current stimulation (tDCS) can boost the beneficial effects of motor rehabilitation, with the boost lasting for at least 3 months post-training [12]. Also, bihemispheric tDCS stimulation (anodal stimulation to excite the ipsilateral side and cathodal stimulation to downregulate the contralateral side) in combination with physical or occupational therapy has been shown to provide a significant improvement in motor function (as measured by Fugl-Meyer and Wolf Motor Function) compared to a sham group [13]. Further, a recent meta-analysis of randomized-controlled trials comparing different forms of tDCS shows that cathodal tDCS is a promising treatment option to improve ADL capacity in people with stroke [14]. Compared to transcutaneous magnetic stimulation (TMS), tDCS devices are inexpensive and easier to operate. Improvement in upper limb motor function can appear after only five tDCS sessions [15], and there are no reports of serious adverse events when tDCS has been used in human trials for periods of less than 40 min at amplitudes of less than 4 mA [16].

Moreover, tDCS stimulation task also seems beneficial for other impairments commonly seen in people post-stroke. Stimulation with tDCS applied for 20 sessions of 30 min over a 4-week period has been shown to decrease depression and improve quality of life in people after a stroke [17, 18]. Four tDCS sessions for 10 min applied over the primary and sensory cortex in eight patients with sensory impairments more than 10 months post-stroke enhanced tactile discriminative performance [19]. Breathing exercises with tDCS stimulation seems to be more effective than without stimulation in patient with chronic stroke [20], and tDCS has shown promise in treating central post-stroke pain [21]. Finally, preliminary research on the effect of tDCS combined with training on resting-state functional connectivity shows promise to better understand the mechanisms behind inter-subject variability regarding tDCS stimulation [22].

Motor functional outcomes in stroke have declined at discharge from inpatient rehabilitation facilities [23, 24], likely a result of the pressures to reduce the length of stay at inpatient rehabilitation facilities as part of a changing and increasingly complex health care climate [25, 26]. Researchers, clinicians, and administrators continue to search for solutions to facilitate and post-stroke rehabilitation after discharge. Specifically, there has been considerable interest in low-cost stroke therapies than can be administered in the home with only a modest level of supervision by clinical professionals.

Home telerehabilitation is a strategy in which rehabilitation in the patient’s home is guided remotely by the therapist using telecommunication technology. If patients can safely apply tDCS to themselves at home, combining telerehabilitation with tDCS would be an easy way to boost therapy without costly therapeutic face-to-face supervision. For people with multiple sclerosis, the study of Charvet et al. (2017) provided tDCS combined with cognitive training, delivered through home telerehabilitation, and demonstrated greater improvement on cognitive measures compared to those who received just the cognitive training [27]. The authors demonstrated the feasibility of remotely supervised, at-home tDCS and established a protocol for safe and reliable delivery of tDCS for clinical studies [28]. Some evidence shows that telerehabilitation approaches are comparable to conventional rehabilitation in improving activities of daily living and motor function for stroke survivors [29, 30], and that telemedicine for stroke is cost-effective [31, 32]. A study in 99 people with stroke receiving training using telerehabilitation (either with home exercise program or robot assisted therapy with home program) demonstrated significant improvements in quality of life and depression [33].

A recent search of the literature suggests that to date, no studies combine tDCS with repetitive tracking training in a home telerehabilitation setting to determine whether the combination leads to improved motor rehabilitation in people with stroke. Therefore, the aim of this pilot project was to explore the safety, usability and feasibility of the combined system. For the tDCS treatment, we used a bihemispheric montage with cathodal tDCS stimulation to suppress the unaffected hemisphere in order to promote stroke recovery [34–37]. For the repetitive tracking training therapy, we used a finger tracking task that targets dexterity because 70% of people post-stroke are unable to use their hand with full effectiveness after stroke [38]. Safety was assessed by noting any decline of 2 points or more in the cognitive testing that persists over more than 3 days. We expect day to day variations of 1 digit. Motor decline is defined by a decline of 6 blocks on the Box and Block test due to muscle weakness. This is based on the minimal detectable change (5.5 blocks/min) [10]. The standard error of measurement is at least 2 blocks for the paretic and stronger side. We expect possible variations in muscle tone that could influence the scoring of the test. Usability was assessed through a questionnaire and by observing whether the participant, under remote supervision, could don the apparatus and complete the therapy sessions. Our intent was to set the stage for a future clinical trial to determine the efficacy of this approach.

## Methods

### Participants

Participants were recruited from a database of people with chronic stroke who had volunteered for previous post-stroke motor therapy research studies at the University of Minnesota. Inclusion criteria were: at least 6 months post-stroke; at least 10 degrees of active flexion and extension motion at the index finger; awareness of tactile sensation on the scalp; and a score of greater than or equal to 24 (normal cognition) on the Mini-Mental State Examination (MMSE) to be cognitively able to understand instructions to don and use the devices [39]. We excluded those who had a seizure within past 2 years, carried implanted medical devices incompatible with tDCS, were pregnant, had non-dental metal in the head or were not able to understand instructions on how to don and use the devices. The study was approved by the University of Minnesota IRB and all enrolled participants consented to be in the study.

### Apparatus

tDCS was applied using the StarStim Home Research Kit (NeuroElectrics, Barcelona, Spain). The StarStim system consists of a Neoprene head cap with marked positions for electrode placement, a wireless cap-mounted stimulator and a laptop control computer. Saline-soaked, 5 cm diameter sponge electrodes were used. For electrode placement, we followed a bihemispheric montage [14] involving cathodal stimulation on the unaffected hemisphere with the anode positioned at C3 and the cathode at C4 for participants with left hemisphere stroke, and vice versa for participants with right hemisphere stroke. Stimulation protocols were set by the investigator on a web-based application that communicated with the tDCS control computer. A remote access application (TeamViewer) was also installed on the control computer, as was a video conferencing application (Skype).

**Fig. 1:**
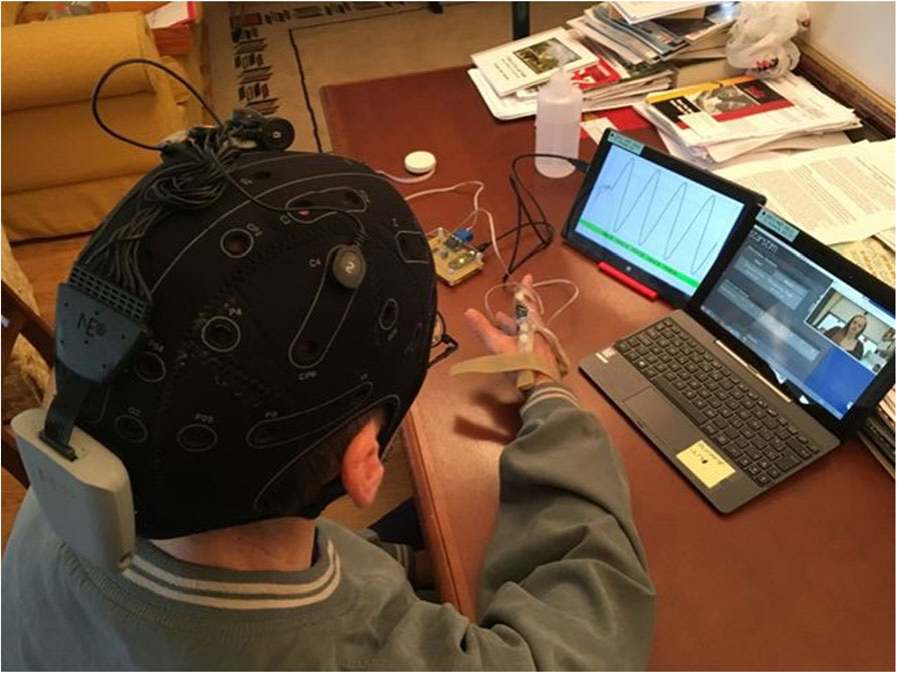
Participant with right hemiparesis receiving transcranial direct current magnetic stimulation (tDCS) in their home simultaneous while performing the finger movement tracking task on the tracking computer (left). The tDCS computer (right) shows the supervising investigator, located off-site, who communicated with the participant through the video conferencing application, controlled the tDCS stimulator through web-based software, and controlled the tracking protocols. (Permission was obtained from the participant for the publication of this picture)

The repetitive finger tracking training system was a copy of what we used in our previous stroke studies [7–9]. The apparatus included an angle sensor mounted to a lightweight brace and aligned with the metacarpophalangeal (MCP) joint of the index finger, a sensor signal conditioning circuit, and a target tracking application loaded on a table computer. Figure 1 shows a participant using the apparatus during a treatment session.

### Assessment measures

We collected demographic information (age, sex, and distance to the university) for each participant. Finger and wrist flexor spasticity was measured with the Modified Ashworth Scale [40], cognitive impairment with the MMSE, physical impairment with the Upper Extremity Fugl-Meyer score [41], and handedness prior to stroke with the Edinburgh Handedness Inventory [42]. The assessments were conducted by physical therapists who were experienced in the proper application of the measurement instruments.

Motor function was assessed before and after treatment using a 60 s trial of the Box and Block Test [43], which assesses rapid grasp and release of single blocks. Cognitive function was assessed before and after treatment using the Digit Span Forward Test [44], which quantifies the largest sequence of numbers the participant can repeat without errors after being recited by the investigator.

Adverse effects of the tDCS were monitored by asking participants whether they experienced any of the following symptoms since the preceding treatment: scalp pain, headache, neck pain, dental pain, tingling, nausea, itching, burning sensation, skin redness, open lesion on skin, abnormal sleep, anxiety, difficulty concentrating, dizziness, impaired memory, altered mood, altered balance, impaired use of the strong hand, or any other problem [45].

Motor function was measured before the first treatment session and after the last treatment session. Cognitive function and adverse effects were measured before each treatment session so that in the event of a cognitive decline or a report of adverse effects, further sessions would be withheld immediately.

Usability was measured by through a post-treatment questionnaire and by observing over the video link the participant interacting with the apparatus.

### Protocol

For this study we followed a protocol that aligned with the guidelines for remote tDCS application suggested by Chavret et al. [46]. These include: (1) training of staff in tDCS treatment and supervision; (2) assessment of the user’s capability to participate in tDCS remotely; (3) ongoing training procedures and materials including assessments of the user and/or caregiver; (4) simple and fail-safe electrode preparation techniques and tDCS headgear; (5) strict dose control for each session; (6) ongoing monitoring to quantify compliance (device preparation, electrode saturation/placement, stimulation protocol), with corresponding corrective steps as required; (7) monitoring for treatment-emergent adverse effects; (8) procedures for discontinuation of a session or study participation including emergency failsafe procedures tailored to the treatment population’s level of need. We included Guidelines 1–3 and 8 in our protocol and had questionnaires and procedures in place to identify any potential adverse events and discontinue any session in case of adverse events prior or during the session. We trained patients to address Guideline 4; the dose (Guideline 5) was controlled by the therapist; we established ongoing monitoring (Guidelines 6–7) for compliance and potential adverse events.

Our study had two scenarios for treatment sessions. Under the first scenario, treatment sessions took place at the university with the supervising investigator in one room and the participant in a separate room to simulate the condition of being at home. We did this with the first 3 participants to be close to emergency services should there be any adverse effects. Under the second scenario, the investigator was at the university and the participant was at home. For both scenarios, the investigator and participant communicated through the video conferencing application. The investigator controlled the treatment applications through the remote access application. Under both scenarios, a second investigator, the observer, was with the participant at all times. The role of the observer was to monitor for adverse events and provide immediate assistance if needed. All instruction and communication with the participant was done by investigator to accurately represent the conditions of a future home-based clinical treatment session.

**Fig. 2:**
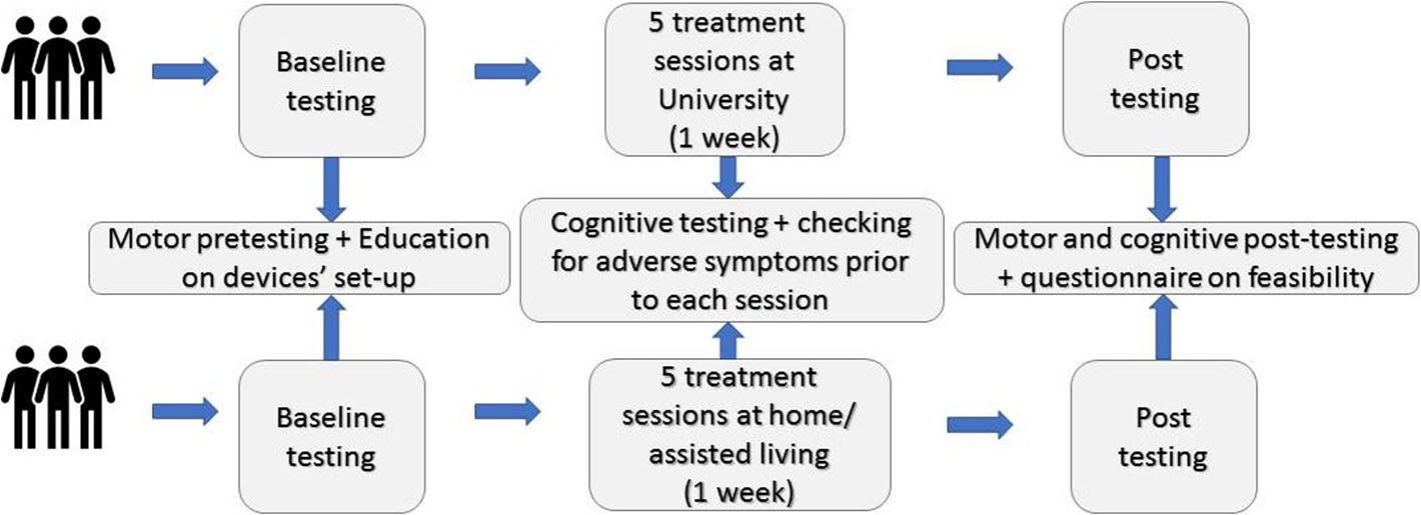
Research design and participants’ study timeline

Figure 2 illustrates the study design and timeline. The initial in-person session involved baseline testing followed by training in how to use the tDCS and tracking training apparatus. The training included how to don the cap so that the tDCS electrodes ended up in the correct locations. The investigator first found and marked the reference point Cz (International 10/20 system for locating scalp electrodes) on the participant’s head by determining the intersection of the line between the nasion and the inion and the line connecting the left and right auricular. The cap was donned and adjusted so that the marked Cz hole in the cap was aligned with the Cz mark. The distance between the front edge of the cap and the eyebrows was noted and this served as the indicator that the cap was positioned properly in future sessions, as monitored by the participant and by the investigator.

The participant was trained to soak the sponge electrodes with saline and secure them in the C3 and C4 marked holes in the cap. Saline-soaked sponge electrodes are standard for tDCS. Sponges were wet but not dripping. The color-coded lead wires were then attached so that the anode was positioned at C3 and the cathode at C4 for participants with left hemisphere stroke, and vice versa for participants with right hemisphere stroke. During the treatment sessions, the participant was guided through these steps by the investigator over the video conferencing application. Additionally, we asked the patient whether they felt some discomfort at the location of the sponges. A caretaker was called upon to look at the head and screen for redness. If the patient lived alone, the patient checked the scalp with a mirror or through taking a picture on their phone, and then inspecting the picture. The participant was also trained in how to apply the hand brace containing the angle sensor to the paretic hand so that the sensor was centered at the lateral side of MCP joint of their index finger. The complete startup procedure was repeated until the participant could re-apply these devices independently with remote guidance, if necessary, from the investigator.

Participants then had 5 treatment sessions either at the university (Fig. 2, top row) or at home (Fig. 2, bottom row). As much as possible, these 5 treatments occurred over consecutive weekdays. The investigator initiated the treatment session by calling the participant’s cell phone. The investigator first conducted the Digit Span Forward Test and the survey of symptoms over the phone. The investigator then instructed the participant to turn on the tDCS control computer and the tracking training tablet computer. Once both computers were connected to the internet, the investigator could gain control using the remote access application and could communicate with the participant via video.

Next, the investigator screened the scalp for possible redness or lesion from the previous treatment by having the participant lower their head to be in view of the computer’s camera and adjust their hair to give a better view of the scalp. The investigator gave further instructions until they were satisfied that all relevant parts of the scalp were assessed. As a safety check, the observer, who was in the same room as the participant, also checked the scalp. The person in the room was only an observer; not a participant. The scalp check did not influence the end result.

The participant then prepared the electrodes and donned the head cap. The investigator viewed the cap position using the video link and if needed guided the participant to make any adjustments. The investigator then remotely active the tDCS software to conduct an impedance check, and if the impedance was too high, coached the participant through taking off the cap, re-wetting the electrodes and putting the cap back on. The impedance check is automatically done by the StarStim system with the threshold set at approximately 10 kohm*.* Once the impedance check was passed, the investigator initiated the tDCS treatment, which was 20 min at 1.5 mA, including a 30 s ramp-up and ramp-down [13].

The investigator then shifted the participant’s attention to donning the finger angle sensor and tablet running the tracking training application. The investigator initiated the application remotely and for approximately 20 min, while simultaneously receiving tDCS, the participant repeatedly extended and flexed the paretic index finger to move the computer-screen cursor as accurately as possible along various target tracks (Fig. 1). Each tracking trial was 5 to 20 s and the investigator remotely adjusted the parameters, including waveform (round, pointy or square waves), frequency (number of waves), amplitude (height of waves), polarity (how high up or down the waves went), and trial duration (how fast the cursor went), to keep the participant challenged and motivated. At the end of each trial, the screen displayed a performance score, related to an accuracy index [47], which provided further motivation to improve tracking accuracy.

At the end of 20 min of tDCS and tracking training therapy, the investigator guided the participant in removing the cap, and then checked the scalp for irritation. The observer also inspected the scalp. The investigator then thanked the participant who powered down the computers and stowed the apparatus, concluding the session for that day. Following the five treatment sessions, the participant returned to the university for the Box and Block and Digit Span Forwards post-treatment tests.

## Results

Six people with chronic stroke participated in the study (3 women; mean (standard deviation, SD) age 61 (10) years; mean (SD) time post-stroke 5.5 (6.5) years; 5 with left hemiplegia due to ischemic stroke; 1 with right hemiplegia due to hemorrhagic stroke.) Table 1 shows the demographic data and stroke characteristics for each participant. The first three participants had their treatment sessions at the university while the second three had their treatment sessions at home. Participants lived between 5 and 20 miles from the university.

**Table 1:** Participant Stroke Characteristics and Treatment Information

ID	Age (years)	Sex	Stroke (years)	Stroke Type and Location	Hemi side	Edinb.	UEFM score	MAS score	MMSE score
1	46	F	0.75	Ischemic, BG, insula, posteroinferior FL, TL	Left	Right	31	2	30
2	60	F	18	Ischemic, MCA	Left	Mixed	38	2	30
3	67	M	6	Ischemic, lacunar infarct of posterior lentiform nucleus, CR	Left	Right	45	1	30
4	70	M	1.3	Ischemic, MCA	Left	Right	48	1	29
5	72	F	2	Ischemic, CR	Left	Right	51	1	29
6	51	M	5	Hemorrhagic, Thalamus, BS	Right	Right	55	0	29

*BG* Basal ganglia, *BS* brain stem, *CR* corona radiata, *Edinb* Edinburgh Handedness Inventory; *F* female, *FL* frontal love, *Hemi side* hemiplegic side, *M* male, *MCA* middle cerebral artery distribution, *UEFM* Upper Extremity Fugl-Meyer, *MAS* Modified Ashworth Scale, *MMSE* Mini-Mental State Examination, *TL* temporal lobe

All six participants completed five sessions (30 sessions total). Table 2 shows the Box and Block pre- and post-test results for the paretic and non-paretic hands, and the pre- and post-test Digit Span Forward test results; as well as the day-to day variations in the Digit Span Forward test.

**Table 2:** Cognitive and motor scores pre- and post-treatment and adverse symptoms reported before each session; and daily Digit Span testing prior to tDCS stimulation

ID	Paretic Hand Box and Block Test (number of blocks)		Non-paretic Hand Box and Block Test (number of blocks)		Digit Span Forward Test (number of digits)					Adverse Symptoms Reported
	Baseline	Posttest	Baseline	Posttest	Baseline		Posttest			
1	4	3	70	73	15		14			None
			Daily Digit Span test		15	13	14	14	14	
2	10	5	50	49	6		6			None
			Daily Digit Span test		6	7	8	7	6	
3	17	21	52	56	9		9			None
			Daily Digit Span test		9	9	6	9	9	
4	15	19	52	55	8		11			None
			Daily Digit Span test		8	9	9	9	11	
5	27	28	72	71	10		10			None
			Daily Digit Span test		10	10	11	11	10	
6	36	38	67	78	12		15			None
			Daily Digit Span test		12	12	10	13	15	
Mean (SD)	18.17 (11.62)	19.00 (13.40)	60.05 (10.19)	63.67 (11.79)	10.00 (3.16)		10.83 (3.31)			
% improvement - Total Group		4.59%		5.23%			8.33%			
- Treatment responders (#3–6)		11.58%		7.00%			15.38%			

There were no meaningful changes in motor or cognitive function except for Participant 2 who showed a 50% reduction in the paretic hand Box and Block Test at posttest. When questioned 2 days after the post-test, the participant stated that their spasticity typically varies throughout the day, and happened to be stronger than usual at the post-test assessment, which worsened their finger dexterity. The participant did not attribute the decrement to the tDCS and felt that their finger dexterity was at its typical level later that day.

Over the five sessions, no participants reported adverse symptoms before or after the treatment, except for the brief, mild, tingly sensation at the electrode sites at the beginning of each treatment in all but one patient who had thick hair. A temporary feeling of tingling is expected. Neither the investigator nor the observer detected reddening of the scalp in any session.

All participants were able to don the cap and adjust to the proper location. After the initial training, they received an illustration showing the proper location of the electrodes on the cap and the cap on the head. During the initial training, landmarks (e.g. cap right above the eye brows on a particular skin line) were given to the patient. After this initial training, no prompting was needed. The observer was only in the room to ensure safety and intervention with donning and doffing the cap was not needed for any session.

From the post-treatment usability questionnaire (Table 3), 5 of the 6 participants found the set-up easy, all six were comfortable with the devices, and all 6 would recommend the telerehabilitation program to others. One participant reported difficulty in setting up the equipment and being uncomfortable in working with computer technology. This participant had good cognitive ability and did not score the lowest in motor function. The same participant, however, would recommend the treatment therapy to others and thought that the treatment was feasible.

**Table 3:** Feasibility questionnaire: Summary of the participants’ responses

1. 1. How difficult/easy was it for you to set up the equipment project?	Very Difficult	Difficult	Neutral	Easy	Very Easy
	0	1	0	4	1
2. How comfortable were you at the beginning in working with computer technology?	Very uncomfortable	Uncomfortable	Neutral	Comfortable	Very Comfortable
	0	1	0	0	5
3. 1. How comfortable are you now in working with computer technology for your rehabilitation?	Very uncomfortable	Uncomfortable	Neutral	Comfortable	Very Comfortable
	0	1	0	0	5
4. 1. To what extent would you recommend tDCS with telerehabilitation to another person with stroke?	Definitely Not Recommend	Not Recommend	Neutral	Recommend	Definitely Recommend
	0	0	0	2	4
5. 1. What is your overall opinion on the feasibility (i.e. ease and capability) of using tDCS with telerehabilitation?	Definitely Not Feasible	Doubtfully Feasible	Neutral	Somewhat Feasible	Highly Feasible
	0	0	0	1	5

Number in each cell indicates number of participants selecting that response

The investigator was able to guide all of the treatment sessions without major incidents. On some occasions, the internet connection was disrupted. When this happened, the participant reconnected the computers and the trial was repeated. Temporary loss of internet connectivity occurred in eight of 30 sessions, and included Participants 1 and 3 at the university and Participant 5 who used the guest network in the community room of their assisted living facility. This happened during the setup phase only; the actual treatment phase was never affected. When internet connectivity problems occurred, the participants could always re-establish the connection after one to three attempts.

A high impedance error trigger occurred in six of the 30 treatments. The impedance check happens before the tDCS treatment is initiated, and the program would not start or continue unless safe impedance was assured. Re-wetting the electrodes solved this problem in each instance. Multiple wetting of the sponges was only needed for one participant who had thick hair.

## Discussion

This study explored the safety and feasibility of applying tDCS in combination with a finger tracking task through telerehabilitation in people with stroke. The results showed no adverse events attributable to tDCS, and all participants successfully completed the five treatment sessions. The role of the observer was minimal, as there were no safety concerns, although the presence of the observer may have influenced the participant to complete all five sessions. As shown by the results of the usability questionnaire and by the observations of the investigator, participants found the apparatus relatively easy to use, including donning the head cap and the finger sensor.

Temporary loss of internet connectivity was the main difficulty encountered; a problem that can be avoided by embedded a cellular interface into the device for internet access or by restricting the therapy to those with reliable internet connections. While Skype was used for this study, future trials will use a secure conferencing application such as VSee Messenger. A second problem was the occasional high impedance error. This is normal for electrotherapy treatments and with proper training, users can learn how to fix the error by re-soaking the electrodes with saline.

Three different electrode montages are possible for tDCS in stroke: anodal in the affected hemisphere; cathodal in the unaffected hemisphere; or combined anodal/cathodal (bihemispheric) [3, 48]. We chose the cathodal stimulation in the unaffected hemisphere to correct the interhemispheric imbalance after stroke by suppressing over-activation in the unaffected hemisphere. While safety with tDCS has been demonstrated with intensity levels up to 4 mA for less than 40 min [49, 50], the choice location of the anode and cathode has been varied over different studies [14]. Cathodal tDCS, however seems to be the most promising treatment option to improve ADL capacity in people with stroke [14].

Variations among participants, age, the time and type of stroke lesion, as well as type of training delivered in conjunction with tDCS, all can influence the effectiveness of tDCS therapy [15, 48, 51, 52]. Rabadi and Aston (2017) demonstrated large effect size improvements in motor function in eight participants with severe motor impairments after acute stroke after applying tDCS for 30 min with 3 h of inpatient rehabilitation therapy, compared to a control group who received sham tDCS and therapy [53]. Several studies have demonstrated retained improved motor abilities between 3 weeks and 3 months post-intervention in chronic stroke after a treatment that combined physical therapy with tDCS stimulation [12, 54, 55].

Unlike transcranial magnetic stimulation (TMS), tDCS technology is simple and potentially low-cost, even for cloud-connected versions. Devices for tDCS therapy have been commercialized and prices should continue to drop if tDCS therapy is adopted as a standard of care. Further, with proper training in the use of the device and occasional remote check-ins with a therapist, we believe that supervised home use of tDCS will be safe, feasible and affordable.

Telerehabilitation offers the opportunity for practice at home as well as reaching people who live remotely. Although geographic data are not available for rehabilitation services, some data are available regarding stroke centers and stroke consultations. For example, in rural areas in northeastern states only 44% of the population had access to stroke centers within 30 miles, compared to 92.3% of the population in urban areas. These states are already implementing telemedicine-enabled stroke consultation [56]. In Minnesota, physical therapists can use telemedicine in real-time or as store-and-forward system for patients under Medicaid. Doing exercises at home with regular therapist check-ins will likely increase adherence to a home exercise therapy program, and may improve the chance of improved outcomes in motor function post-stroke. Another advantage of telerehabilitation is the time and cost saved for the patient not having to drive to the rehabilitation center, pay for parking, and for one-on-one therapy services. This savings is especially relevant for patients living in rural and remote communities. With telerehabilitation, the clinic also realizes savings by reducing costs associated with room set-up, intake, supplies, and therapist time beyond that associated with the remote check-in.

Participants in this study neither declined nor improved in motor and cognitive function. Because the long-term goal is to use tDCS plus tracking training as a post-stroke therapy tool, efficacy must be demonstrated, which means showing improved motor function when compared to tracking training alone. The reason no improved in motor function occurred in this study is it is likely that each treatment session was too short and there were an insufficient number of treatment sessions. Previous finger tracking therapy studies showed improved motor skills after 18 to 20 treatment sessions, each 45 to 60 min, a far more intensive paradigm than what we used for this study [7–9]. Combining tDCS stimulation with motor learning therapy, especially one that targets precise finger control, may enhance its impact on functional recovery. Recently, more robot and virtual reality games have provided gloves or other devices for these hand and finger movements to be trained more intensively [57, 58]. Clinical trials should use multivariate models capturing different patient baseline characteristics to predict which patients would respond to treatment. This will help develop a targeted, individualized brain stimulation therapy for patients with stroke [59]. For those studies, the minimum selection criteria for participants who could benefit from the therapy would be the ability to don the tDCS electrodes and finger tracker hardware either themselves or with the aid of a caregiver, and the cognitive ability to remember the treatment procedure and safety steps. The ability to reliably place the electrodes in their proper location will depend on the particular tDCS system chosen for the study. Additionally, participants must have a minimum ability to move the finger, which for this study was set at 10 degrees, but could be set lower for future studies. In our study, the participants were cognitively high functioning, but according to Woytowicz et al. (2017), our patients are categorized with moderate to mild upper limb impairments, with visible limitations in hand use [60]. The reason that patients with severe upper limb impairment were not included is because one of our inclusion criteria stated that the patients had to be able to move at least 10 degrees with the index finger in order to perform the finger tracking movement. Therefore, we cannot generalize our findings to the total population. Further studies should investigate if patients without hand function on the affected side are able to don the tDCS cap. Our age range of patients is 46–72 years of age, with average age 61 years of age. Recent evidence suggests that 1/3 of the people with a stroke are less than 65 years old [61], but our results may not generalize to the general population of individuals with stroke.

## Conclusions

As in-clinic healthcare costs continue to rise, economical, home-based treatments for post-stroke motor rehabilitation will become increasingly significant. Based on the results of this study, a home-based post-stroke therapy that combines low-cost tDCS and tracking training can be a safe treatment option. Although the study sample size was small, the participants found the set-up easy, were comfortable with the devices and unanimously recommended the use of tDCS and finger tracking as a telerehabilitation program. We conclude that tDCS combined with finger tracking training is safe and feasible for the people with stroke. Clinical trials are needed to determine if this promising remote therapy with tDCS and finger tracking is effective.

## Abbreviations

MCP: metacarpophalangeal
MMSE: Mini-Mental State Examination
SD: standard deviation
tDCS: transcranial direct current stimulation
